# The *Shigella flexneri* Type 3 Secretion System Is Required for Tyrosine Kinase-Dependent Protrusion Resolution, and Vacuole Escape during Bacterial Dissemination

**DOI:** 10.1371/journal.pone.0112738

**Published:** 2014-11-18

**Authors:** Carole J. Kuehl, Ana-Maria Dragoi, Hervé Agaisse

**Affiliations:** Department of Microbial Pathogenesis, Yale School of Medicine, Boyer Center for Molecular Medicine, New Haven, Connecticut, United States of America; Rockefeller University, United States of America

## Abstract

*Shigella flexneri* is a human pathogen that triggers its own entry into intestinal cells and escapes primary vacuoles to gain access to the cytosolic compartment. As cytosolic and motile bacteria encounter the cell cortex, they spread from cell to cell through formation of membrane protrusions that resolve into secondary vacuoles in adjacent cells. Here, we examined the roles of the Type 3 Secretion System (T3SS) in *S. flexneri* dissemination in HT-29 intestinal cells infected with the serotype 2a strain 2457T. We generated a 2457T strain defective in the expression of MxiG, a central component of the T3SS needle apparatus. As expected, the Δ*mxiG* strain was severely affected in its ability to invade HT-29 cells, and expression of *mxiG* under the control of an arabinose inducible expression system (Δ*mxiG/pmxiG*) restored full infectivity. In this experimental system, removal of the inducer after the invasion steps (Δ*mxiG/pmxiG* (Ara withdrawal)) led to normal actin-based motility in the cytosol of HT-29 cells. However, the time spent in protrusions until vacuole formation was significantly increased. Moreover, the number of formed protrusions that failed to resolve into vacuoles was also increased. Accordingly, the Δ*mxiG/pmxiG* (Ara withdrawal) strain failed to trigger tyrosine phosphorylation in membrane protrusions, a signaling event that is required for the resolution of protrusions into vacuoles. Finally, the Δ*mxiG/pmxiG* (Ara withdrawal) strain failed to escape from the formed secondary vacuoles, as previously reported in non-intestinal cells. Thus, the T3SS system displays multiple roles in *S. flexneri* dissemination in intestinal cells, including the tyrosine kinase signaling-dependent resolution of membrane protrusions into secondary vacuoles, and the escape from the formed secondary vacuoles.

## Introduction

The intracellular pathogen *Shigella flexneri* is the causative agent of bacillary dysentery in humans [1]. *S. flexneri* enters the cells of the human colon by triggering its own engulfment, leading to the formation of a primary vacuoles [2]. The pathogen rapidly escapes the formed vacuoles and gains access to the cytosolic compartment where it multiplies and displays actin-based motility [3], [4]. This process of invasion and escape from the primary vacuole relies on the expression of the bacterial Type 3 Secretion System (T3SS), a needle-like apparatus that translocates T3SS effector proteins directly from the bacterial cytoplasm into the cytoplasm of the eukaryotic cell [2], [5]. Once *S. flexneri* is free in the cytoplasm of the primary infected cell, it begins the process of dissemination by first displaying actin-based motility. As motile bacteria encounter the plasma membrane, they form protrusions that project into adjacent cells and resolve into secondary vacuoles [6]. The bacteria once again escape these vacuolar compartments, gaining access to the cytosol of adjacent cells where they continue the dissemination process. Mutations in IcsA, a central component of actin-based motility, or VirB, a master transcriptional regulator of *S. flexneri* virulence gene expression, result in the inability to complete this cycle of cell-to-cell spread and drastically decrease the virulence of the corresponding mutant strains [7], [8].

The *S. flexneri* T3SS displays 25-30 known secreted effectors [9]. Some of these secreted effectors and the Type 3 Secretion apparatus itself are necessary for invasion of the primary infected cell. In order to study the pathogenic defects caused by mutations in these genes essential to pathogenesis, conditional mutants are used. The conditional mutants are strains with insertions or deletions in the gene of interest, complemented with plasmids harboring the gene of interest expressed from an inducible promoter, such as the arabinose-inducible pBAD expression system. The gene of interest is expressed for a short period of time to allow for invasion of the primary infected cell; then the inducer (eg. Arabinose) is removed for the remainder of the infection to allow the uncomplemented mutant to proceed in cell-to-cell spread [10]. Using such an inducible complementation system, deletion of the T3SS translocases (IpaA, IpaB, IpaC, IpaD) as well as structural components of the T3SS (MxiM, Spa33, MxiG), or transcriptional regulators of the T3SS (VirF, VirB), has been shown by electron microscopy to lead to an accumulation of bacteria in secondary vacuoles [10], [11]. These results have established that the T3SS is not only necessary for invasion of the primary infected cell and escape of the primary vacuole, but is also involved in escape of *S. flexneri* from the secondary vacuole.

During host cell invasion, pre-translated effectors are secreted upon entry when the T3SS tip complex composed of the effector proteins IpaB and IpaD senses the plasma membrane [12] leading first to secretion of the pore-forming translocases IpaB and IpaC, followed by other early effectors. Once IpaB and IpaC are secreted, their freed chaperone, IpgC, acts as a co-activator of transcription with MxiE, which leads to expression of a second subset of effectors with promoters that contain a MxiE-box motif [13], [14]. MxiE transcriptional regulation is further de-repressed by the secretion of OspD1, which inhibits MxiE activation until OspD1 is secreted with the first round of pre-translated effectors upon T3SS activation [15]. The tight control of MxiE activation and MxiE-regulated gene expression make MxiE-regulated genes a good reporter system for activation of the *S. flexneri* T3SS.

Using *lacZ*-fusions to MxiE-regulated genes *virA* and *ipaH7.8*, and using a GFP-fusion to *ipaH7.8*, Demers *et al.* and Campbell-Valois *et al.* respectively demonstrated an initial increase in MxiE-regulated gene expression in cytosolic *S. flexneri* followed by a decrease in gene expression [5], [16]. The work by Campbell-Valois *et al.* proceeded further to demonstrate the re-initiation of the *S. flexneri* T3SS by detecting *ipaH7.8-*GFP expressing bacteria in protrusions prior to secondary vacuole formation [16].

Much of the previous work studying defects in *S. flexneri* dissemination as an indicator of virulence has been done in the non-intestinal HeLa cell line [11], [17]–[19]. HeLa cells allow for *S. flexneri* invasion, cytosolic motility, and protrusion formation, but have a very low frequency of protrusion resolution into secondary vacuoles as compared to the colonic epithelial cell line HT-29 [20]. This low frequency of resolution is partly due to a defect in tyrosine kinase signaling in protrusions, essential to vacuole formation in HT-29 cells [20].

In this work, we compared the cell-to-cell spread of wild-type 2457T *S. flexneri* serotype 2a in HT-29 cell monolayers to the cell-to-cell spread of a Δ*mxiG* mutant, which lacks a central component of the T3SS needle apparatus. By tracking the dissemination of individual bacteria, we uncovered multiple roles for the T3SS in the cell-to-cell spread of *S. flexneri* in intestinal cells, including the escape from secondary vacuoles, as previously reported in non-intestinal cells, and the tyrosine kinase signaling-dependent resolution of protrusions into vacuoles.

## Materials and Methods

### Cell lines and bacterial strains

HT-29 cells (ATCC #HTB-38) were cultured at 37°C in McCoy's 5A medium (GIBCO) supplemented with 10% Fetal bovine serum (Invitrogen). The *Shigella flexneri* strain used in this study is serotype 2a strain 2457T [21]. The Δ*mxiG* mutant was created in the 2457T strain via lambda red recombination using the method of Datsenko and Wanner [22] with homologous recombination primers MxiG5′ATCTATAGACGCTATTTGCGGATAGCAGCAGAGAGCAAGCAGAATAATCGAAGGATATAACATATGAATATCCTCCTTAG and MxiG3′ATAATGAACTCAATGTCCAATCATCATTCGGTACTGTAACACTCATTTTATCCTCACTTAGTGTAGGCTGGAGCTGCTTC. The Δ*mxiG* strain was complemented by expressing wild-type *mxiG* from the arabinose-inducible pBAD promoter in vector pBAD18 (ATCC #87393).

### Bacterial infection and invasion assays

Overnight cultures of *S. flexneri* harboring a plasmid with an IPTG-inducible CFP expression system were grown in LB broth at 37°C with shaking, then diluted 1∶100 in LB broth and grown to exponential phase (approximately 3 hours) at 37°C with shaking. The media was removed from HT-29 cells prior to infection with *S. flexneri* and replaced with fresh media. L-Arabinose was added to the media at 0.2% (final) for experiments with the Δ*mxiG* strain complemented with pBAD*mxiG* (p*mxiG*). Infection was synchronized by centrifuging the plate or live-cell dish at 1,000 rpm for 5 minutes. Bacteria were allowed to invade the monolayer for 30 minutes at 37°C before the media was removed and replaced with fresh media for all strains along with gentamicin at 50 µM (final) to kill extracellular bacteria. One hour before infections were stopped, or one hour before live imaging began, IPTG at 4 mM (final) was added to the medium to induce CFP expression. To visually assess invasion, and for actin tail quantification, infected HT-29 cell monolayers were incubated 4 or 2 hours, respectively, at 37°C then fixed in PBS with 4% formaldehyde at 25°C for 20 minutes before immuno-staining. For the foci size analysis infected HT-29 cell monolayers were incubated for 8 hours at 37°C.

### Invasion assay

To determine the invasiveness of individual *S. flexneri* strains, exponential-phase cultures of *S. flexneri* 2457T, Δ*mxiG*, and Δ*mxiG*/p*mxiG* +0.2% arabinose each harboring a plasmid carrying chloramphenicol resistance, were diluted in McCoy's 5A medium and used to infect confluent monolayers of HT-29 cells grown on glass coverslips. Serial dilutions of the tested strains diluted in McCoy's 5A media were plated on LB agar plus chloramphenicol (10 µg/ml) to determine input CFUs. The infections were synchronized by centrifuging 24-well infection dishes at 1000 rpm for 5 minutes. After 30 minutes of infection at 37°C, the monolayers were washed four times with PBS and fresh McCoy's 5A media plus gentamicin 50 µM (final) was added to kill extracellular bacteria. At two hours post infection, the monolayers were again washed four times with PBS to remove extracellular bacteria and then incubated for 10 minutes at 37°C in 0.5 ml 0.1% Triton X-100 in PBS to lyse the eukaryotic cells. Lysed HT-29 cells were washed from the coverslips by pipetting up and down. Serial dilutions of the lysate from each infected monolayer were plated on LB agar plus chloramphenicol (10 µg/ml) to determine intracellular CFUs compared to the input value determined for each strain.

### Immunofluorescence and antibodies

Cells were fixed with 4% formaldehyde in PBS for 20 minutes at 25°C, then washed and permeabilized with 0.5% Triton X-100 in PBS for staining with rabbit polyclonal anti-*Shigella* (1∶300, Viro-Stat), or permeabilized with 0.5% Triton X-100 in PBS for staining with anti-phospho tyrosine (1∶500, Cell Signaling) antibody, and Alexa Fluor Phalloidin (1∶1000, Invitrogen). The secondary antibodies used were anti-rabbit Alexa 594, and anti-mouse Alexa 594 (1∶1000, Molecular Probes).

### Size of infection foci

The size of CFP-expressing *S. flexneri* infection foci formed in HT-29 cell monolayers was determined in 384-well plate format as described previously [20]. After fixation and DAPI staining, the plates were imaged using a TE2000 automated microscope (Nikon) equipped with a motorized stage (Prior), motorized filter wheels (Sutter Instrument, Inc.) and a 10× objective (Nikon) mounted on a piezo focus drive system (Physik Instrumente). Image acquisition was conducted using the Metamorph 7.1 Software (Molecular Devices, Inc.). For image analysis, high intensity pixels corresponding to the bacteria were selected against the low intensity pixels corresponding to the background levels by using the threshold function of the MetaMorph 7.1 imaging software. Selected pixels were clustered by proximity using the “close” morphological filter in order to create a single object that represented a given focus of infection. The integrated morphometry analysis (IMA) module was used to determine the area of each infection focus in a given image. Image analyses were conducted on at least 100 infection foci in three independent experiments.

### Cytosolic velocity and dynamics of cell-to-cell spread

Time-lapse microscopy was performed as described previously [20]. HT-29 cells were grown on 35-mm imaging dishes at 37°C in 5% CO_2_ (MatTek, Ashland, MA). Cells were infected with *S. flexneri* and imaged with a Nikon TE2000 spinning disc confocal microscope driven by the Volocity software package (Improvision). For analysis of cytosolic velocity, Z-stacks were captured every 30 seconds for at least 5 minutes and individual bacteria were tracked in four dimensions using the tracking module of the Volocity software. The average speed was calculated for bacteria undergoing motility, defined as>0.01 µm/s for at least 90 consecutive seconds.

For analysis of protrusion and vacuole formation, and vacuole escape, Z-stacks were captured 2.5 hours post infection every 5 minutes for at least 180 minutes. For clarity in presenting representative images, the Z planes corresponding to the basal plasma membrane were subsequently excluded from the merged Z-stacks. Protrusions were defined as plasma membrane extensions that formed as a result of motile bacteria reaching the plasma membrane at the border between two cells and progressing further into adjacent cells. Vacuoles were defined as membrane bound compartments that derived from protrusions and displayed a continuous lining of the plasma membrane surrounding the bacteria. Vacuoles were no longer connected to the sending cell via a membranous neck. Free bacteria were defined as bacteria that were previously observed in vacuoles, but were no longer surrounded by a continuous lining of the plasma membrane.

## Results

### Generation of the Δ*mxiG* strain 2457T

**Figure 1:**
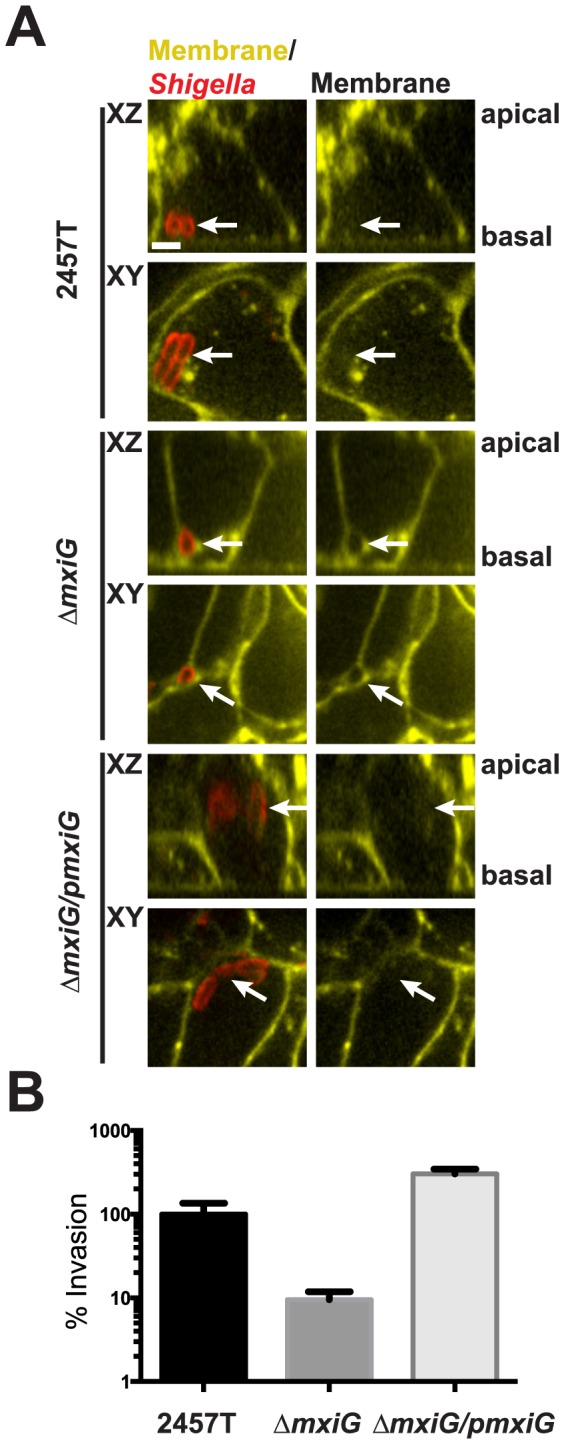
Quantification of *S. flexneri* invasion of HT-29 cell monolayer. (A) Representative images (XY and XZ planes) of HT-29 cells expressing plasma membrane-targeted YFP at 4 h post infection with the wild type strain 2457T, the isogenic Δ*mxiG* strain and the complemented Δ*mxiG/pmxiG*. Bacteria (red) were stained with an anti-*Shigella* antibody. Arrows indicate the presence or absence of host cell membrane surrounding the bacteria. Scale bar, 2.5 µm. (B) Percent invasion of HT-29 cell monolayers with wild-type 2457T strain set to 100%. Values represent the mean +/−SD of three independent experiments. Statistical analysis; ** p = 0.0095, **** p<0.0001, unpaired t test.

To study the roles of the Type 3 Secretion System (T3SS) in the dissemination of *S. flexneri* serotype 2a strain 2457T in HT-29 intestinal cells, we generated a mutant defective in the expression of MxiG, an essential component of the needle apparatus. The *mxiG* deletion was generated using the lambda red method of Datsenko and Wanner [22]. The chloramphenicol cassette was removed and the deletion was complemented by introduction a wild-type copy of *mxiG* encoded on pBAD24 (Figure S1). The T3SS is necessary for *S. flexneri* to trigger its own entry into non-phagocytic cells. To allow for invasion of the HT-29 cell monolayer, *mxiG* was expressed from the pBAD promoter by induction with 0.2% arabinose. After 30 minutes, the cells were washed and fresh media containing gentamicin but no arabinose was added to kill extracellular bacteria and repress the p*mxiG* expression, respectively. Representative images of infected HT-29 cells 4 hours post infection are shown in Figure 1A. Both the wild-type 2457T strain and the complemented Δ*mxiG/pmxiG* isogenic strain invaded the HT-29 monolayer, escaped from the primary vacuoles and gained access to the cytosolic compartment, as determined by confocal microscopy of cells expressing plasma membrane-targeted YFP (Figure 1A). By contrast, the Δ*mxiG* strain located in between adjacent HT-29 cells, but was not observed free in the cytosolic compartment, suggesting that the Δ*mxiG* strain was defective for invasion and/or primary vacuole escape in polarized HT-29 cells (Figure 1A). We also quantified the invasion properties of the 2457T strain and isogenic Δ*mxiG* strains in HT-29 cells by using a standard gentamicin protection assay at 2 hours post infection, where the wild-type invasion rate was set to 100%. In this assay, the Δ*mxiG* strain was ten-fold less invasive than the wild-type strain (Figure 1B, Δ*mxiG*). By contrast, the complemented *ΔmxiG* strain (Δ*mxiG/pmxiG*) consistently invaded the HT-29 cell monolayer at three-fold the level of the 2457T strain (Figure 1B, Δ*mxiG/pmxiG*). These results show that the inducible expression of MxiG rescued the invasion defects observed with the Δ*mxiG* strain.

### Quantification of cytosolic actin-based motility

**Figure 2:**
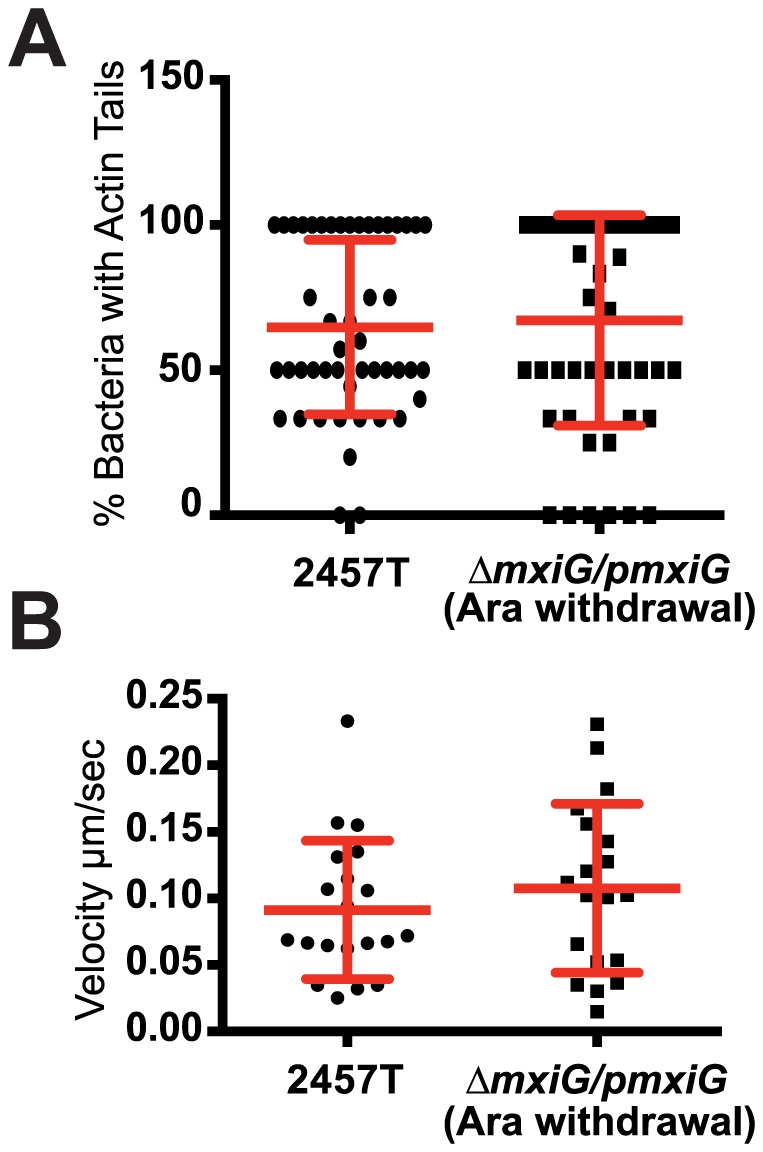
Quantification of intracellular motility. (A and B) Infection of HT-29 cells with CFP-expressing wild type and Δ*mxiG/pmxiG* (Ara withdrawal) strains. (A) Graph showing the percent of bacteria with actin tail in the cytoplasm of HT-29 cells 2 h post infection. Points represent the percent of bacteria with actin tail per infected cell. Red bars indicate the mean +/- SD of three independent experiments. Statistical analysis: p = 0.73. (B) Graph showing the velocity of wild type and Δ*mxiG/pmxiG* (Ara withdrawal) bacteria in the cytoplasm of HT-29 cells 2 h post infection. Points represent the average velocity of individual bacteria. Red bars indicate the mean +/−SD of three independent experiments. Statistical analysis: p = 0.39, unpaired t test.

We have previously shown that the intestinal HT-29 cell line provides an excellent system for modeling *S. flexneri* actin-based motility [23]. We used the arabinose-inducible MxiG expression system to investigate whether the T3SS was required for actin tail formation in the cytosol of HT-29 cells (Figure S2). We determined the percent of cytosolic bacteria displaying actin tails 2.5 hours post-infection (2 hours after removal of arabinose from the tissue culture medium to repress expression of *mxiG* from the pBAD promoter). As shown in Figure 2A, the levels of wild-type and Δ*mxiG* strain displaying actin tails were not significantly different. We also recorded the velocity of motile bacteria in the cytosol (defined as maintaining a velocity>0.01 µm/s for at least 90 seconds) and found no significant difference between the wild-type and the Δ*mxiG/pmxiG* (Ara withdrawal) strain (Figure 2B). Finally, we tracked motile bacteria between the 3 and 4 hour time points and determined that 97.5% of wild-type and 75% and Δ*mxiG/pmxiG* (Ara withdrawal) strains encountered the plasma membrane and formed protrusions, respectively. The slight decrease in the percentage of Δ*mxiG/pmxiG* (Ara withdrawal) bacteria that formed protrusions compared to the wild-type strain was due to mutant bacteria that did not sustain actin-based motility as consistently as the wild-type strain.

### Quantification of the Δ*mixG* strain dissemination in HT-29 cells

**Figure 3:**
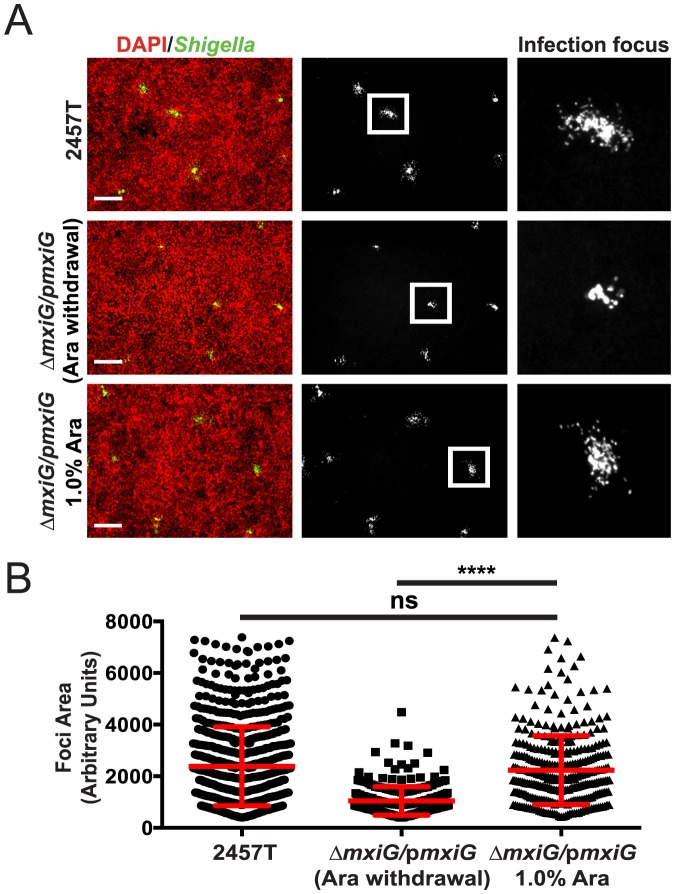
Quantification of *S. flexneri* dissemination in HT-29 cells. (A,B) Infection of HT-29 cells with CFP-expressing *S. flexneri*. (A) Representative images showing the size of infection foci 8 h post infection comparing wild-type 2457T to the Δ*mxiG* strain complemented with p*mxiG* with 1.0% arabinose removed after the initial 30 minutes of infection (Δ*mxiG/pmxiG* (Ara withdrawal)), and the Δ*mxiG* mutant complemented with p*mxiG* with 1.0% arabinose throughout infection (Δ*mxiG/pmxiG* 1.0% Ara). Green, *Shigella*; red, DNA. Scale bar, 200 µm. (B) Computer-assisted image analysis was used to quantify the size of the infection foci and the average focus size was determined. Individual points represent individual foci, red bars indicate the mean +/−SD. Statistical analysis; ****, p<0.0001, unpaired t-test.

We have previously shown that the intestinal HT-29 cell line provides an excellent system for modeling *S. flexneri* cell-to-cell spread [20], [23]. In this model system, motile bacteria spread from cell-to-cell, which results in the formation of typical infection foci eight hours post infection (Figure 3A). We compared the area of wild-type 2457T foci with the complemented Δ*mxiG* strain foci under two conditions: when 1.0% arabinose was removed after 30 minutes of invasion (Δ*mxiG/pmxiG* (Ara withdrawal)), and when 1.0% arabinose induction remained throughout the eight-hour infection period (Δ*mxiG/pmxiG* 1.0% Ara). As shown in Figure 3A, the wild-type strain formed large foci of infection. By contrast, the Δ*mxiG/pmxiG* (Ara withdrawal) strain formed much smaller foci (Figure 3A, Ara withdrawal). This spreading defect was rescued by addition of 1.0% Arabinose (Figure 3A, 1% Ara). To quantify the spreading efficiency of the wild-type, the complemented and the uncomplemented Δ*mxiG* strains, we used computer-assisted image analysis to determine the area of individual infection foci (Figure 3B). The Δ*mxiG/pmxiG* (Ara withdrawal) mean foci area was significantly smaller than wild-type 2457T, reflecting a striking spreading defect in cells infected with the T3SS-defective mutant. The Δ*mxiG/pmxiG* 1.0% Ara mean foci area was significantly larger than the Δ*mxiG/pmxiG* (Ara withdrawal) mean foci area, and not significantly different from the wild-type mean foci area, showing that 1.0% Arabinose induction of MxiG expression in the Δ*mxiG* strain fully rescued the observed spreading defect (Figure 3B).

### Dynamics of protrusion and vacuole formation of the wild-type 2457T strain

**Figure 4:**
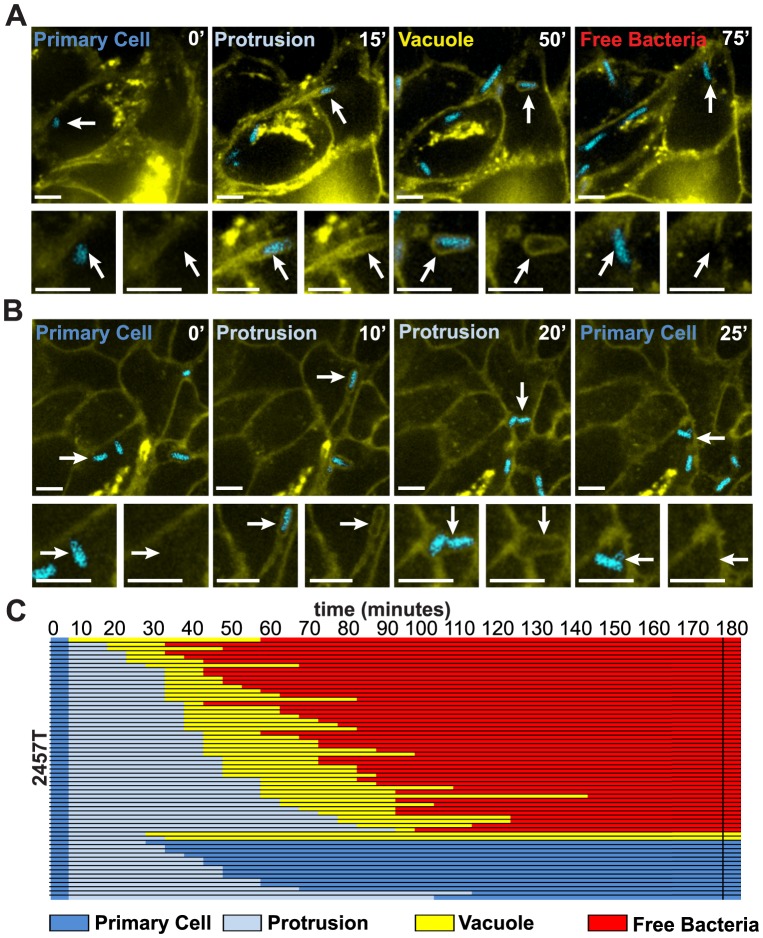
Dynamics of wild-type 2457T dissemination in HT-29 cells. (A–C) Time-lapse microscopy of plasma membrane-targeted YFP-expressing HT-29 cells infected with CFP-expressing wild-type strain 2757T. Yellow, plasma membrane; Cyan, *Shigella*. (A,B) Representative images showing the progression of a single bacterium over time. For each panel, the top image shows a low magnification image of infected cells and the bottom images show an enlargement of the tracked bacterium (merged bacterium and membrane channels, left; membrane channel only, right). (A) Successful progression of a bacterium (white arrows, top panel) from the primary cell cytoplasm (0′, Primary Cell), into a membrane protrusion (15′, Protrusion) that resolve into a secondary vacuole (50′, Vacuole) from which the bacterium escape and gains access to the cytoplasm of the adjacent cell (75′, Free bacteria). (B) Unsuccessful progression of a bacterium (white arrows, top panel) from the primary cell cytoplasm (0′, Primary Cell) into a membrane protrusion (10′, Protrusion) that retracted towards the primary infected cell (20′, Protrusion) and returned the bacterium to the cytosol of the primary infected cell (25′, Primary Cell). (C) Tracking analysis of 60 bacteria, which formed protrusions in 20 independent foci. All bacteria were tracked for at least 180 minutes and the progression of the dissemination process was depicted using the color key shown at the bottom of panel C. Primary cell, dark blue; Protrusion, light blue; Vacuole, yellow; Free bacteria in adjacent cell, red. Scale bars, 5 µm.

To further investigate the role of the T3SS in *S. flexneri* dissemination, we used time-lapse confocal microscopy to determine the sequence and timing of events leading to spread from cell to cell. To this end, we used HT-29 cell monolayers expressing membrane-targeted YFP infected with the wild-type 2457T strain expressing IPTG-inducible CFP. In order to track individual bacteria, we used a low multiplicity of infection resulting in the presence of 2-5 bacteria in the cytosol of infected cells 2.5 hours post-infection. As shown in Figure 4A and video S1, when cytosolic and motile bacteria encountered the plasma membrane, they formed protrusions that elongated into adjacent cells. The resolution of protrusions led to the formation of vacuoles from which the pathogen escaped and gained access to the cytosol of adjacent cells. In some instances, the bacteria formed protrusions, but vacuole formation failed. In these instances, the protrusions collapsed, bringing the pathogen back to the primary infected cell (Figure 4B and video S2).

In Figure 4C, we showed the spreading progression of 60 wild-type bacteria as they emerged from the primary infected cells and projected into adjacent cells. Dark blue shading at the beginning of each track represents the location of the bacterium in the primary infected cell cytosol. Light blue shading indicates the bacterium in protrusions, yellow shading refers to a bacterium in a secondary vacuole, and red shading indicates that the double membrane of the secondary vacuole was no longer visible, showing that the bacterium was free in the cytoplasm of the adjacent cell. Our tracking data established that 75% (45/60) of the wild-type bacteria successfully gained access to the cytosol of adjacent cells (Figure 4C, 180′ red shading, and Figure S3A). Less than 5% (2/60) of the bacteria that formed secondary vacuoles, failed to gain access to the cytosol of adjacent cells within the three-hour time frame of observation (Figure 4C, 180′, yellow shading, and Figure S3A). Of all the bacteria that formed protrusions, less than 25% (13/60) failed to progress into secondary vacuoles. These failed protrusions all collapsed and returned the bacteria back to the cytosol of the primary infected cell (Figure 4C, 180′, dark blue shading, and Figure S3A). On average, the wild-type strain spent 36+/−2.5 minutes in protrusions, before the protrusion resolved into a secondary vacuole, from which the pathogen escaped in 31+/−2 minutes.

### Dynamics of protrusion and vacuole formation of the Δ*mxiG/pmxiG* (Ara withdrawal) strain

**Figure 5:**
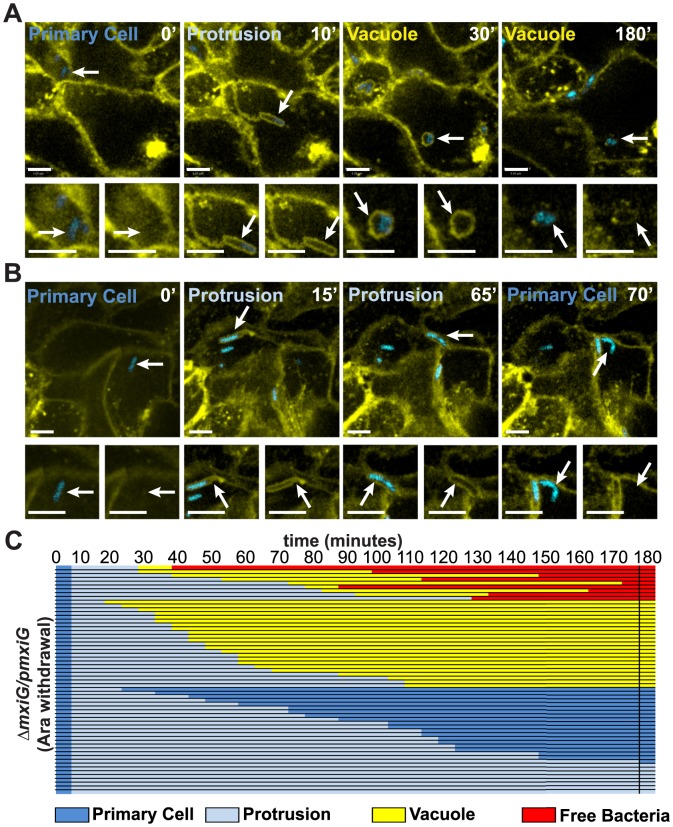
Dynamics of Δ*mxiG* dissemination in HT-29 cells. (A–C) Time-lapse microscopy of plasma membrane-targeted YFP-expressing HT-29 cells infected with the CFP-expressing Δ*mxiG/pmxiG* (Ara withdrawal) strain. Yellow, plasma membrane; Cyan, *Shigella*. (A,B) Representative images showing the progression of a single bacterium over time. For each panel, the top image shows a low magnification image of infected cells and the bottom images show an enlargement of the tracked bacterium (merged bacterium and membrane channels, left; membrane channel only, right). (A) Unsuccessful progression of a bacterium (white arrows, top panel) from the primary cell cytoplasm (0′, Primary Cell) into a membrane protrusion (10′, Protrusion) that resolved into a vacuole (30′, Vacuole) from which the pathogen did not escape (180′, vacuole). Note that the trapped bacterium divided into at least 5 bacteria. (B) Unsuccessful progression of a bacterium (white arrows, top panel) from the primary cell cytoplasm (0′, Primary Cell) into a membrane protrusion (15′, Protrusion) that retracted towards the primary infected cell (65′, Protrusion) and returned the pathogen to the cytosol of the primary infected cell (70′, Primary Cell). (C) Tracking analysis of 60 bacteria, which formed protrusions in 25 independent foci. All bacteria were tracked for at least 180 minutes and the progression of the dissemination process was depicted using the color key shown at the bottom of panel C. Primary cell, dark blue; Protrusion, light blue; Vacuole, yellow; Free bacteria in adjacent cell, red. Scale bars, 5 µm.

We next analyzed the spreading progression of the Δ*mxiG/pmxiG* (Ara withdrawal) strain in HT-29 cells. The initial 30 minutes of infection was in the presence of 0.2% arabinose to induce the expression of *mxiG* from the pBAD promoter, thereby allowing for invasion. The inducer was then removed as we added fresh medium containing gentamicin to kill extracellular bacteria. Figure 5A shows representative images of the spreading progression of the Δ*mxiG/pmxiG* (Ara withdrawal) strain as bacteria emerged from the primary infected cells and formed protrusions that projected into adjacent cells. In contrast with the situation observed with the wild-type strain, a large number of the bacteria formed protrusions that resolved into vacuoles, from which the pathogen never escaped (Figure 5A and video S3). These vacuoles often harbored several bacteria, suggesting that the Δ*mxiG/pmxiG* (Ara withdrawal) strain was trapped, but kept dividing in these membrane-bound compartments (Figure 5A, vacuole, arrowhead). We also observed a large number of bacteria that formed protrusions that collapsed, returning the pathogen back to the primary infected cells (Figure 5B and video S4).

**Figure 6:**
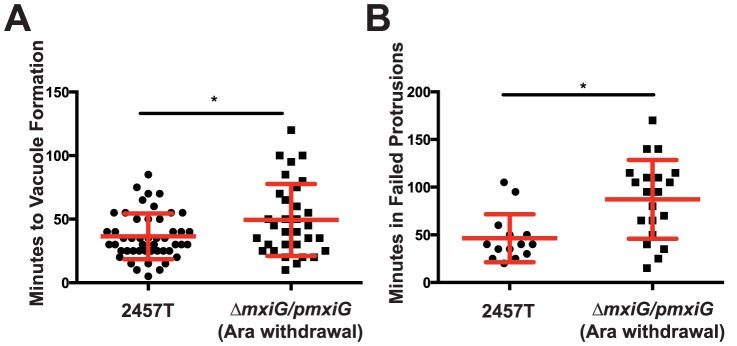
The T3SS is involved in protrusion resolution and vacuole formation. Quantification of the tracking data presented in Figure 4C and 5C. (A) Graph showing the average time spent by the wild-type 2457T and the Δ*mxiG/pmxiG* (Ara withdrawal) strains in protrusions prior to vacuole formation. Statistical analysis, p = 0.0148, unpaired t test. (B) Graph showing the average time spent by the wild-type 2457T and the Δ*mxiG/pmxiG* (Ara withdrawal) strains in the protrusions that extended, collapsed and returned the bacterium to the cytosol of the primary cell. Statistical analysis, p = 0.0025, unpaired t test.

Our tracking data of 60 Δ*mxiG/pmxiG* (Ara withdrawal) bacteria established that ∼40% (23/60) of the bacteria formed vacuoles and did not gain access to the cytosol of adjacent cells (Figure 5C and Figure S3B). 22% (5/23) of these vacuoles resulted from bacteria that divided multiple times within the protrusion, forming large branching protrusions (video S5). We never observed large branching protrusions with the wild-type strain. This high rate of vacuole escape failure compared to the wild-type (3.3%, (2/60)) indicates a role for the T3SS in escape from the secondary vacuole, as previously reported in non-intestinal cells [10], [11]. In addition, ∼45% (28/60) of the Δ*mxiG/pmxiG* (Ara withdrawal) bacteria forming protrusions either stayed in the formed protrusions until the end of the three-hour observation window, or returned to the primary infected cell cytoplasm, indicating a role of the T3SS in the resolution of protrusions into vacuoles (Figure 5C and Figure S3B). We also analyzed the time spent by the bacteria in protrusions. The time to vacuole formation was significantly increased in HT-29 cells infected with the Δ*mxiG/pmxiG* (Ara withdrawal) strain compared to wild-type (Figure 6A). Additionally, the time spent by the Δ*mxiG/pmxiG* (Ara withdrawal) strain in failed protrusions, before returning to the primary infected cell cytosol, was also increased, with an average time of 46+/−7 minutes and 87+/−9 minutes for the wild-type and the Δ*mxiG/pmxiG* (Ara withdrawal) strain, respectively (Figure 6B).

### Tyrosine phosphorylation in protrusions

**Figure 7:**
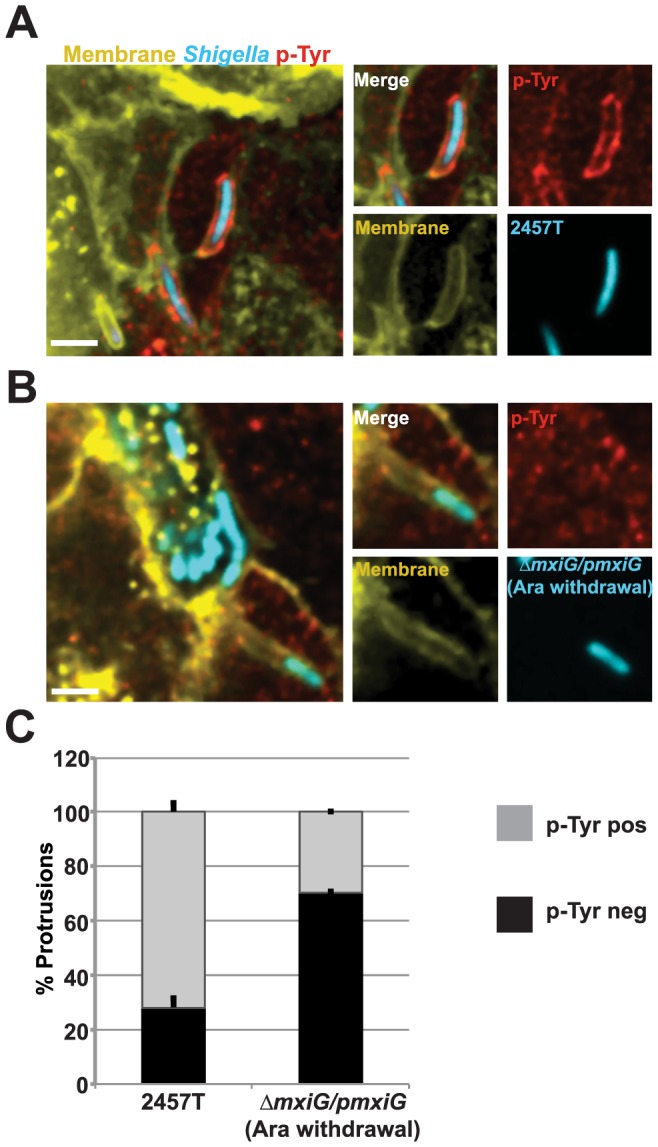
The T3SS is required for activation of tyrosine kinase signaling in protrusions. (A) Representative images of HT-29 cells expressing a YFP-membrane marker (yellow), infected with the CFP-expressing wild-type strain 2457T (cyan) and stained for phospho-tyrosine residues (red). (B) Representative images of HT-29 cells expressing membrane-targeted YFP (yellow), infected with the CFP-expressing Δ*mxiG/pmxiG* (Ara withdrawal) strain (cyan) and stained for phospho-tyrosine residues (red). Scale bar 5 µm. (C) Graph showing the percent of phospho-tyrosine positive and phospho-tyrosine negative protrusions for the wild-type and the Δ*mxiG/pmxiG* (Ara withdrawal) strains. Values indicate the mean +/SD of three independent experiments. Statistical analysis; p-Tyr positive p<0.0001, unpaired t test.

We previously reported that the membrane of the protrusions formed in HT-29 cells is highly enriched in phospho-tyrosine residues, suggesting a role for tyrosine kinase signaling in *S. flexneri* protrusions [20]. Accordingly, we showed that treatment with the tyrosine kinase inhibitor Imatinib led to the formation of phospho-tyrosine negative protrusions, which resulted in severely impaired bacterial dissemination due to an inability to resolve protrusions into vacuoles [20]. To evaluate the potential role of the T3SS in inducing tyrosine kinase signaling in protrusions, we counted the number of protrusions that displayed phospho-tyrosine residues in cells infected with the wild-type strain and the Δ*mxiG/pmxiG* (Ara withdrawal) strain (Figure 7A and 7B). More than 70% of wild-type protrusions displayed phospho-tyrosine staining (Figure 7C). By contrast, less than 30% of Δ*mxiG/pmxiG* (Ara withdrawal) strain protrusions displayed phospho-tyrosine residues (Figure 7C). Thus, the T3SS is required for efficient tyrosine kinase signaling in the protrusions formed by *S. flexneri* in HT-29 cells.

## Discussion

The human pathogen *S. flexneri* displays the ability to invade epithelial cells of the human colon, followed by replication in the cytosolic compartment and dissemination to adjacent cells. Numerous studies conducted in various intestinal as well as non-intestinal cell lines have demonstrated an essential role for the T3SS in the invasive properties of *S. flexneri* [24]–[27]. In addition, a role for the T3SS in escape from the secondary vacuoles formed during the dissemination steps of the infection process has been demonstrated in non-intestinal cells [10], [11]. Here, we conducted comparative analyses of the dissemination process in intestinal cells infected with wild-type *S. flexneri* strain 2457T and the isogenic Δ*mxiG/pmxiG* (Ara withdrawal) strain defective in the T3SS. This experimental system allowed us to establish the role of the T3SS in secondary vacuole escape in intestinal cells. In addition, we uncovered a previously unappreciated role for the T3SS in the resolution of protrusions into secondary vacuoles. Below we discuss the implications of our findings.

### A role for the T3SS in protrusion resolution

*S. flexneri* dissemination relies on its ability to form plasma membrane protrusions as cytosolic and motile bacteria encounter cell-cell junctions. The formed protrusions resolve into secondary vacuoles from which the pathogen escapes to gain access to the cytosol of adjacent cells. Recently, Campbell-Valois *et al*. showed that the *S. flexneri* T3SS is not only active at the sites of invasion, but is also strongly re-activated in the protrusions and to a lesser extent in the subsequent secondary vacuoles formed during the dissemination process [16]. In the present study, we expanded on these observations and showed that the T3SS is functionally required for timely and successful resolution of protrusions into secondary vacuoles. We had previously shown that the resolution process relies on the activation of tyrosine kinase signaling in *S. flexneri* protrusions [20]. However, the exact contribution of the bacterium to these signaling events remained unclear. Our present work demonstrates that the activity of the T3SS is required for activation of tyrosine kinase signaling in protrusions. A role for the T3SS system in triggering tyrosine kinase signaling at the plasma membrane is not unprecedented. In 2009, Mounier *et al.* demonstrated that an *S. flexneri* strain proficient in secretion of effector proteins, but lacking the effector domain of the translocase protein IpaC, failed to activate the non-receptor tyrosine kinase Src at the site of invasion [28]. In the case of the resolution of protrusions into vacuoles, future work will determine whether the effector function of the IpaC translocase is necessary and/or sufficient, or whether the secretion/translocation of other effectors is required to modulate tyrosine kinase signaling in membrane protrusions.

### Assessing the roles of the T3SS using an inducible expression system

Since the T3SS is strictly required for the early steps of infection, the study of its potential role in cell-to-cell spread requires the use of an inducible expression system to generate conditional mutants that would behave as the wild-type strain during the early steps of the infection process, but would then behave as loss-of-function strains after removal of the inducer [10]. It is important to keep in mind that this conditional mutant approach relies on the decreased expression of a functional gene product due to the combined effects of transcriptional shutdown and subsequent protein turnover. Therefore, we may be underestimating the extent of the T3SS phenotype with the arabinose inducible/glucose repressible conditional complementation of the *mxiG* mutation used in this study. Accordingly, our tracking data clearly indicated that a large proportion of the Δ*mxiG/pmxiG* (Ara withdrawal) strain either formed protrusions that never resolved into vacuoles or successfully formed secondary vacuoles from which it never escaped. However, similar to wild-type bacteria, a small proportion of the Δ*mxiG/pmxiG* (Ara withdrawal) bacteria (15%) were able to escape secondary vacuoles and become free bacteria in adjacent cells. These results strongly suggest that these Δ*mxiG* “mutant” bacteria still expressed sufficient levels of MxiG allowing for vacuole formation and escape. Similarly, we may as well underestimate the importance of the T3SS in the resolution process due to the presence of mutant bacteria that expressed levels of MxiG sufficient to form, but not to escape vacuoles. Finally, we note that the percent of cytosolic bacteria displaying actin tails and the recorded cytosolic velocities were similar in HT-29 cells infected with the wild-type and Δ*mxiG/pmxiG* (Ara withdrawal) strains, suggesting that the T3SS does not contribute to actin-based motility. This is in contrast with the conclusions of a previous study by Leung *et al.*, who used a conditional *virB* mutant to suggest a role for the T3SS in the initiation of actin-based motility in the cytosol of HeLa cells [17]. These phenotypic differences may well reflect differences in the experimental systems used to examine the role of the T3SS (VirB vs MxiG). Alternatively, the transcriptional regulator VirB may regulate the expression of virulence proteins required for the bacteria to develop actin-based motility, but in a T3SS-independent manner. In agreement with this notion, several studies indicated that after the initial invasion steps, the T3SS is no longer active in the cytosol of infected cells [5], [16].

## Concluding Remarks

In conclusion, the activity of the T3SS is required for *S. flexneri* dissemination through the resolution of protrusions into secondary vacuoles and the subsequent escape from the formed secondary vacuoles. Future work will be required to determine the identity of the T3SS effectors that mediate these multiple roles of the T3SS. We note that different sets of effectors may be required for protrusions resolution and vacuole escape, perhaps suggesting the existence of regulatory mechanisms controlling the specificity of the T3SS activity in space and time.

## Supporting Information

Figure S1 **Generation of the Δ** ***mxiG*** **strain.** Primers with 5′ ends homologous to the 5′ and 3′ ends of *mxiG*, and 3′ ends homologous to the chloramphenicol acetyl transferase (CAT) cassette were used to amplify the CAT cassette using plasmid pKD3 as template [22]. The CAT cassette was integrated into the large virulence plasmid of the *S. flexneri* strain 2457T at the *mxiG* locus using the lambda red recombinase system [22], creating the Δ*mixG::CAT* strain. To create the Δ*mixG* strain, the Flp recombinase was introduced into the Δ*mixG::CAT* strain with plasmid pCP20 [22] to remove the CAT cassette by recombining the two *flp* sites at the 5′ and 3′ ends of the CAT cassette (yellow). The resulting Δ*mxiG* strain contained the upstream untranslated region of *mxiG*, the remaining *flp* recombination site, and the downstream untranslated region of *mxiG*.
(EPS)

Figure S2 **Representative images of actin tails.** Representative confocal microscopy images showing cytosolic bacteria with actin tails in HT-29 cells. Wild-type 2457T-infected cell (top), Δ*mxiG/pmxiG* (Ara withdrawal)-infected cell (bottom). Yellow, Plasma membrane (Mb-YFP); red F,-actin (phalloidin); blue, *S. flexneri* (CFP).
(EPS)

Figure S3 **The T3SS is involved in vacuole formation and vacuole escape.** Pie charts showing the percentage of tracked bacteria that successfully gained access to the cytosol of adjacent cells (red, Free bacteria), failed to escape secondary vacuoles (yellow, Vacuole failure), or formed protrusions that did not resolve into vacuoles (blue, Protrusion failure) in HT-29 cells infected with the wild-type strain 2457T (A) and the isogenic Δ*mxiG/pmxiG* (Ara withdrawal) strain (B).
(EPS)

Video S1 **Successful dissemination, 2457T.** Time-lapse confocal microscopy of *S. flexneri* 2457T cell-to-cell spread showing successful escape from the double membrane vacuole in an adjacent cell corresponding to selected images of each stage of spreading in Figure 4A. Yellow, Plasma membrane; blue, *S. flexneri*. Time points are separated by 5 minutes.
(MP4)

Video S2 **Protrusion failure, 2457T.** Time-lapse confocal microscopy of *S. flexneri* 2457T cell-to-cell spread showing protrusion failure corresponding to selected images of each stage of spreading in Figure 4B. Yellow, Plasma membrane; blue, *S. flexneri*. Time points are separated by 5 minutes.
(MP4)

Video S3 **Vacuole failure, Δ** ***mxiG/pmxiG*** **(Ara withdrawal).** Time-lapse confocal microscopy of *S. flexneri* Δ*mxiG/pmxiG* (Ara withdrawal) cell-to-cell spread showing vacuole escape failure and multiplication within the double membrane vacuole in an adjacent cell corresponding to selected images of each stage of spreading in Figure 5A. Yellow, plasma membrane; blue, *S. flexneri* Δ*mxiG/pmxiG* (Ara withdrawal). Time points are separated by 5 minutes.
(MP4)

Video S4 **Protrusion failure, Δ** ***mxiG/pmxiG*** **(Ara withdrawal).** Time-lapse confocal microscopy of *S. flexneri* Δ*mxiG/pmxiG* (Ara withdrawal) cell-to-cell spread showing protrusion failure corresponding to selected images of each stage of spreading in Figure 5B. Yellow, plasma membrane; blue, *S. flexneri* Δ*mxiG/pmxiG* (Ara withdrawal). Time points are separated by 5 minutes.
(MP4)

Video S5 **Protrusion branching and vacuole failure, Δ** ***mxiG/pmxiG*** **(Ara withdrawal).** Time-lapse confocal microscopy of *S. flexneri* Δ*mxiG/pmxiG* (Ara withdrawal) cell-to-cell spread showing bacterial division and branching of the protrusion followed by vacuole escape failure and continued bacterial division within the double membrane vacuole in an adjacent cell. Yellow, plasma membrane; blue, *S. flexneri* Δ*mxiG/pmxiG* (Ara withdrawal). Time points are separated by 5 minutes.
(MP4)
